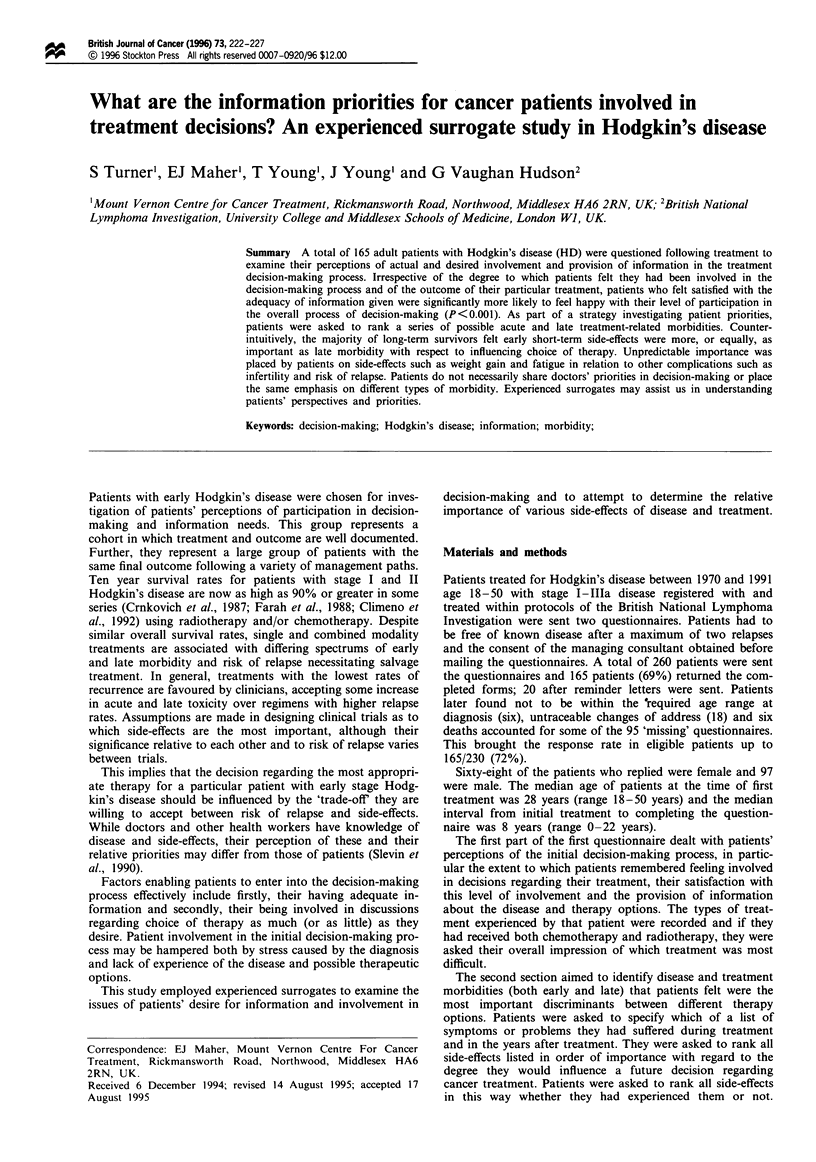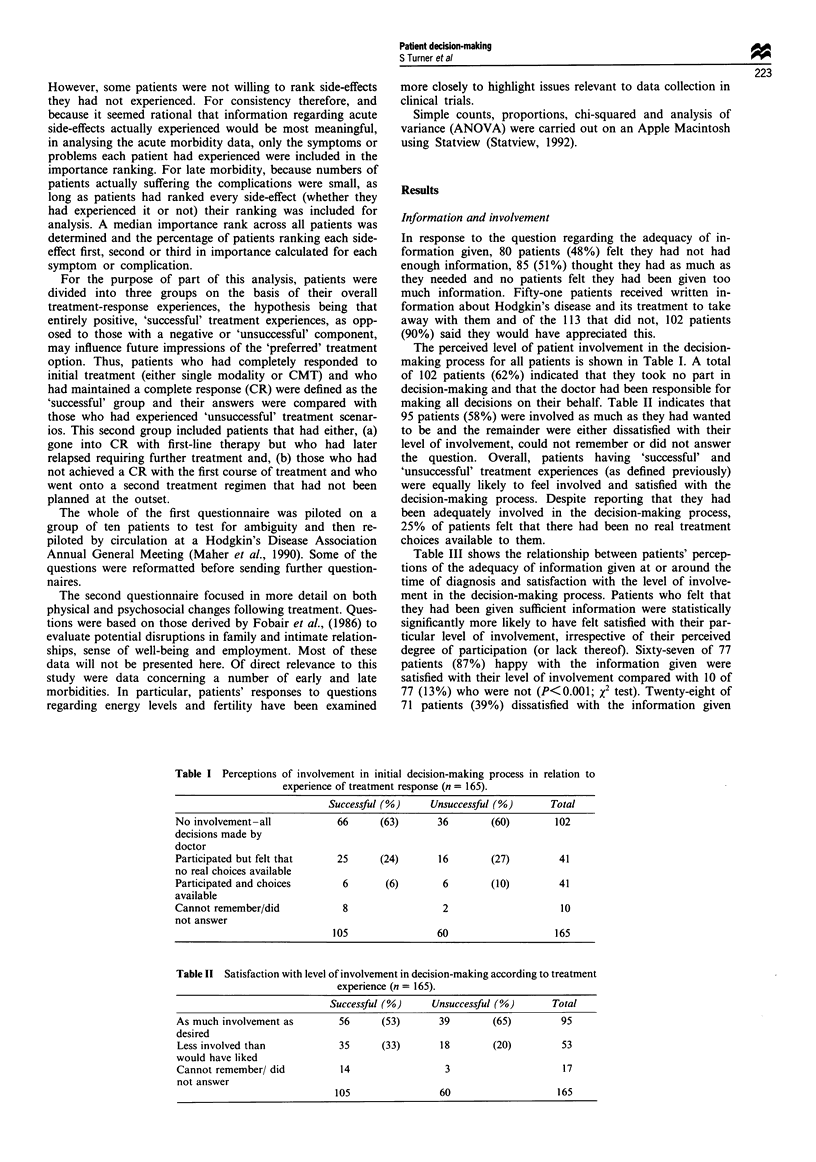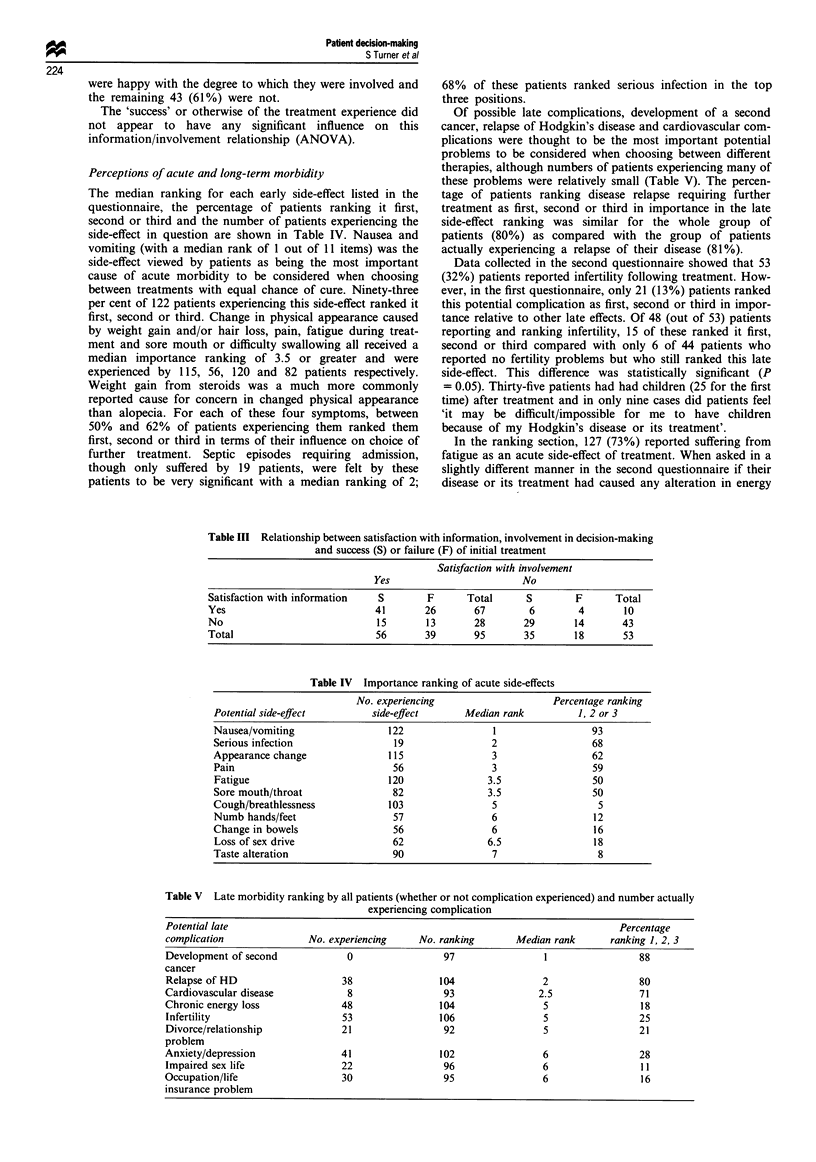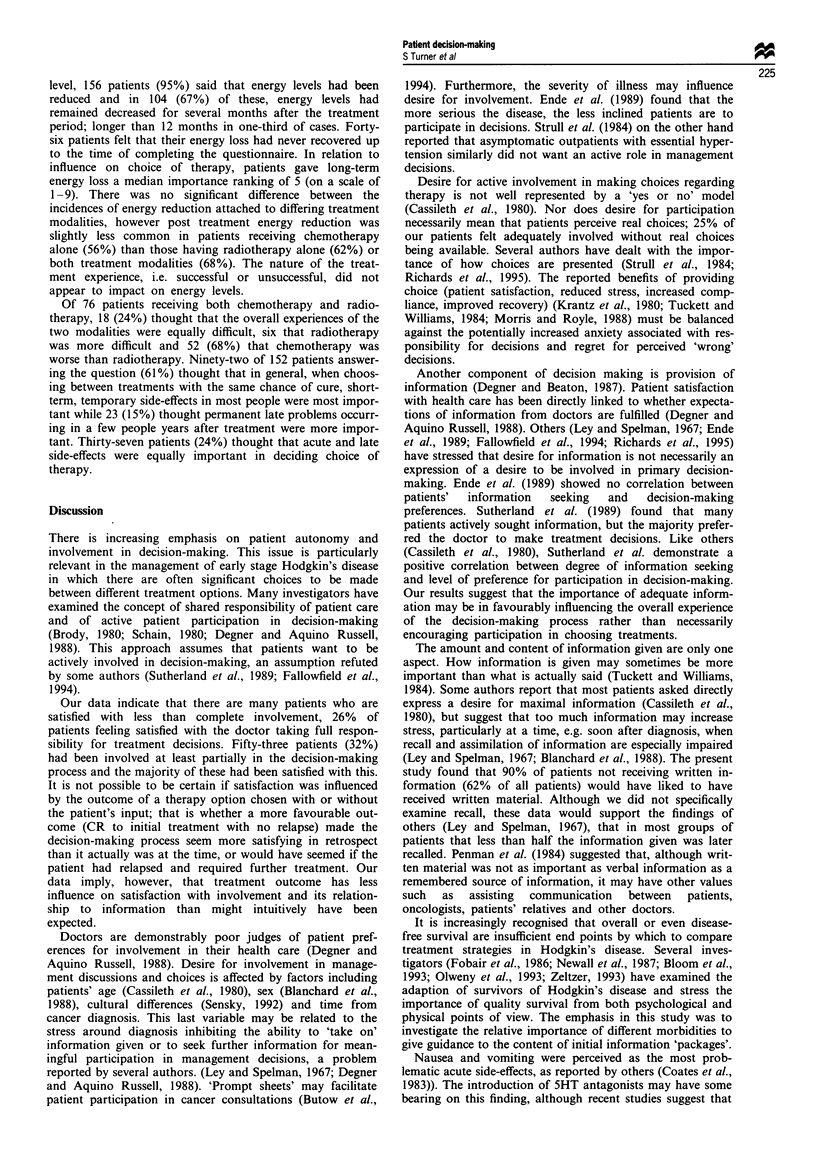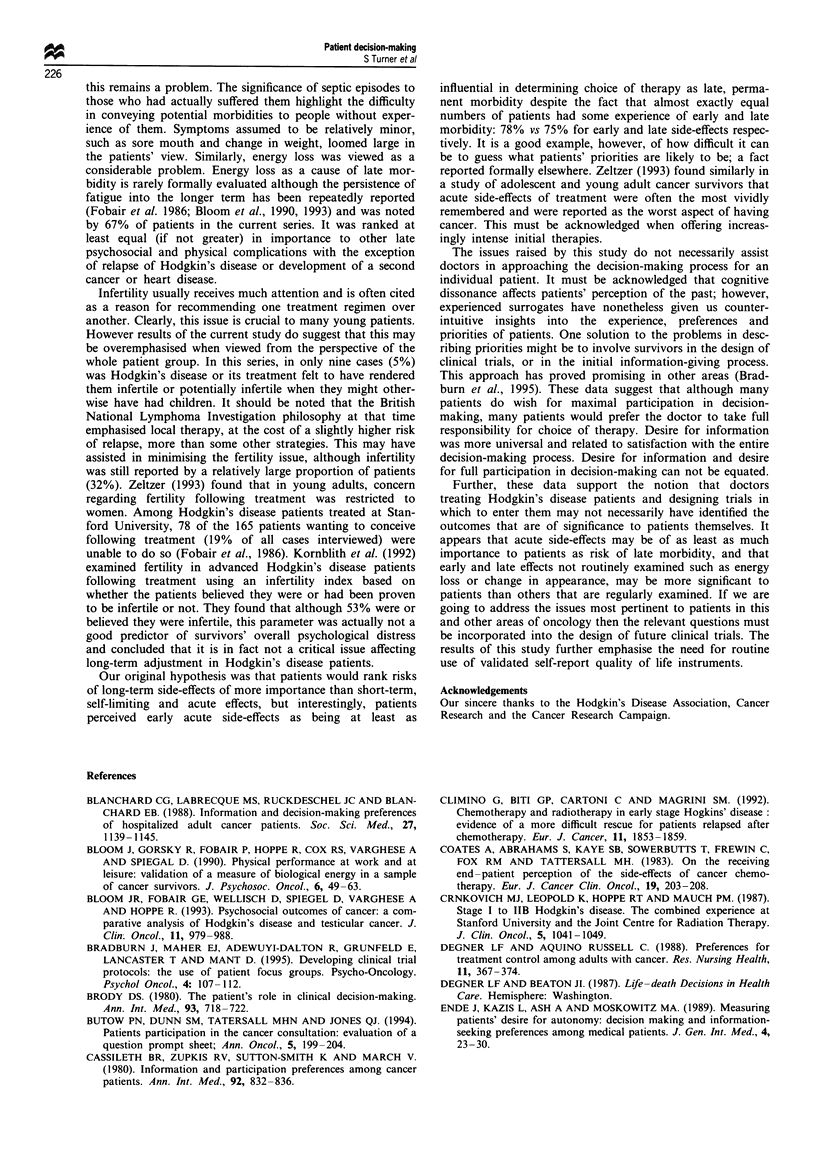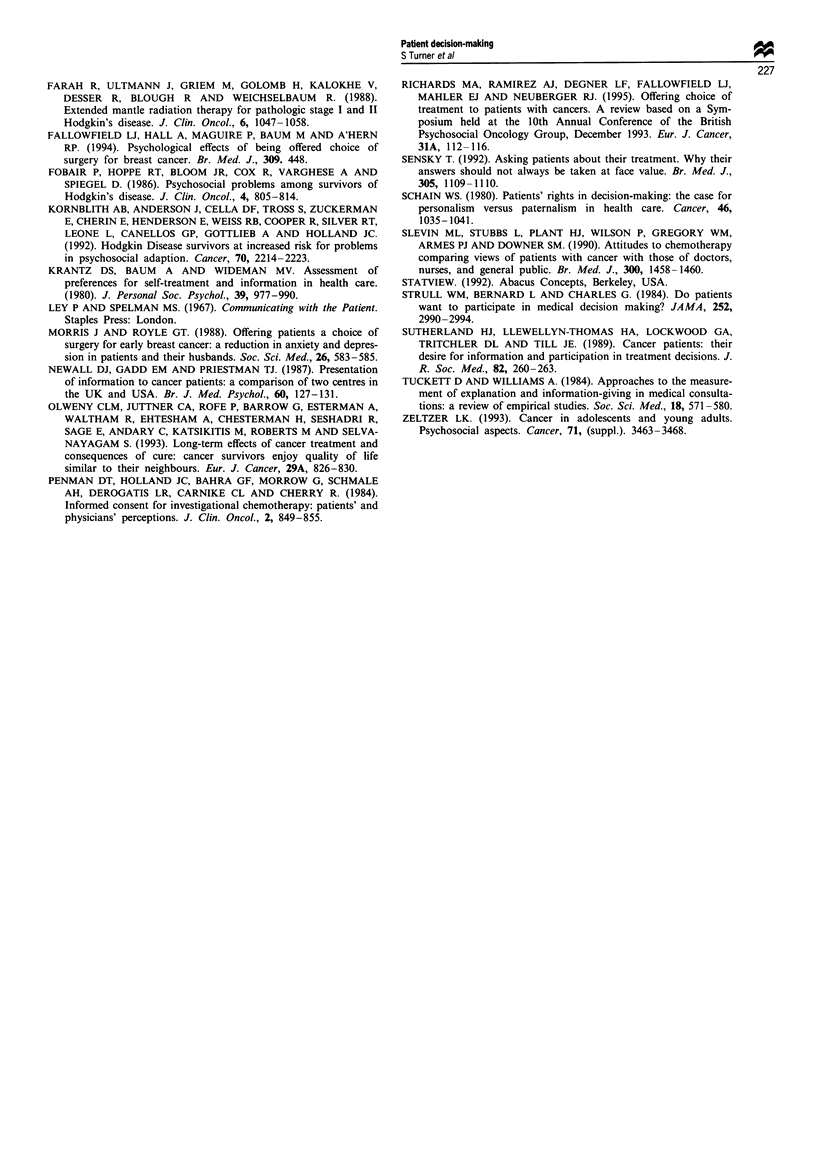# What are the information priorities for cancer patients involved in treatment decisions? An experienced surrogate study in Hodgkin's disease.

**DOI:** 10.1038/bjc.1996.39

**Published:** 1996-01

**Authors:** S. Turner, E. J. Maher, T. Young, J. Young, G. Vaughan Hudson

**Affiliations:** Mount Vernon Centre for Cancer Treatment, Northwood, Middlesex, UK.

## Abstract

A total of 165 adult patients with Hodgkin's disease (HD) were questioned following treatment to examine their perceptions of actual and desired involvement and provision of information in the treatment decision-making process. Irrespective of the degree to which patients felt they had been involved in the decision-making process and of the outcome of their particular treatment, patients who felt satisfied with the adequacy of information given were significantly more likely to feel happy with their level of participation in the overall process of decision-making (P < 0.001). As part of a strategy investigating patient priorities, patients were asked to rank a series of possible acute and late treatment-related morbidities. Counter-intuitively, the majority of long-term survivors felt early short-term side-effects were more, or equally, as important as late morbidity with respect to influencing choice of therapy. Unpredictable importance was placed by patients on side-effects such as weight gain and fatigue in relation to other complications such as infertility and risk of relapse. Patients do not necessarily share doctors' priorities in decision-making or place the same emphasis on different types of morbidity. Experienced surrogates may assist us in understanding patients' perspectives and priorities.


British Journal of Cancer (1996) 73, 222-227
© 1996 Stockton Press All rights reserved 0007-0920/96 $12.00

### What are the information priorities for cancer patients involved in treatment decisions? An experienced surrogate study in Hodgkin's disease

S Turner[1], EJ Maher[1], T Young[1], J Young[1] and G Vaughan Hudson[2]

[1]*Mount Vernon Centre for Cancer Treatment, Rickmansworth Road, Northwood, Middlesex HA6 2RN, UK;* [2]*British National Lymphoma Investigation, University College and Middlesex Schools of Medicine, London W1, UK.*

**Summary** A total of 165 adult patients with Hodgkin's disease (HD) were questioned following treatment to examine their perceptions of actual and desired involvement and provision of information in the treatment decision-making process. Irrespective of the degree to which patients felt they had been involved in the decision-making process and of the outcome of their particular treatment, patients who felt satisfied with the adequacy of information given were significantly more likely to feel happy with their level of participation in the overall process of decision-making ($P<0.001$). As part of a strategy investigating patient priorities, patients were asked to rank a series of possible acute and late treatment-related morbidities. Counter-intuitively, the majority of long-term survivors felt early short-term side-effects were more, or equally, as important as late morbidity with respect to influencing choice of therapy. Unpredictable importance was placed by patients on side-effects such as weight gain and fatigue in relation to other complications such as infertility and risk of relapse. Patients do not necessarily share doctors' priorities in decision-making or place the same emphasis on different types of morbidity. Experienced surrogates may assist us in understanding patients' perspectives and priorities.

**Keywords:** decision-making; Hodgkin's disease; information; morbidity;

Patients with early Hodgkin's disease were chosen for investigation of patients' perceptions of participation in decision-making and information needs. This group represents a cohort in which treatment and outcome are well documented. Further, they represent a large group of patients with the same final outcome following a variety of management paths. Ten year survival rates for patients with stage I and II Hodgkin's disease are now as high as 90% or greater in some series (Crnkovich *et al.*, 1987; Farah *et al.*, 1988; Climeno *et al.*, 1992) using radiotherapy and/or chemotherapy. Despite similar overall survival rates, single and combined modality treatments are associated with differing spectrums of early and late morbidity and risk of relapse necessitating salvage treatment. In general, treatments with the lowest rates of recurrence are favoured by clinicians, accepting some increase in acute and late toxicity over regimens with higher relapse rates. Assumptions are made in designing clinical trials as to which side-effects are the most important, although their significance relative to each other and to risk of relapse varies between trials.

This implies that the decision regarding the most appropriate therapy for a particular patient with early stage Hodgkin's disease should be influenced by the 'trade-off' they are willing to accept between risk of relapse and side-effects. While doctors and other health workers have knowledge of disease and side-effects, their perception of these and their relative priorities may differ from those of patients (Slevin *et al.*, 1990).

Factors enabling patients to enter into the decision-making process effectively include firstly, their having adequate information and secondly, their being involved in discussions regarding choice of therapy as much (or as little) as they desire. Patient involvement in the initial decision-making process may be hampered both by stress caused by the diagnosis and lack of experience of the disease and possible therapeutic options.

This study employed experienced surrogates to examine the issues of patients' desire for information and involvement in decision-making and to attempt to determine the relative importance of various side-effects of disease and treatment.

### Materials and methods

Patients treated for Hodgkin's disease between 1970 and 1991 age 18–50 with stage I–IIIa disease registered with and treated within protocols of the British National Lymphoma Investigation were sent two questionnaires. Patients had to be free of known disease after a maximum of two relapses and the consent of the managing consultant obtained before mailing the questionnaires. A total of 260 patients were sent the questionnaires and 165 patients (69%) returned the completed forms; 20 after reminder letters were sent. Patients later found not to be within the required age range at diagnosis (six), untraceable changes of address (18) and six deaths accounted for some of the 95 'missing' questionnaires. This brought the response rate in eligible patients up to 165/230 (72%).

Sixty-eight of the patients who replied were female and 97 were male. The median age of patients at the time of first treatment was 28 years (range 18–50 years) and the median interval from initial treatment to completing the questionnaire was 8 years (range 0–22 years).

The first part of the first questionnaire dealt with patients' perceptions of the initial decision-making process, in particular the extent to which patients remembered feeling involved in decisions regarding their treatment, their satisfaction with this level of involvement and the provision of information about the disease and therapy options. The types of treatment experienced by that patient were recorded and if they had received both chemotherapy and radiotherapy, they were asked their overall impression of which treatment was most difficult.

The second section aimed to identify disease and treatment morbidities (both early and late) that patients felt were the most important discriminants between different therapy options. Patients were asked to specify which of a list of symptoms or problems they had suffered during treatment and in the years after treatment. They were asked to rank all side-effects listed in order of importance with regard to the degree they would influence a future decision regarding cancer treatment. Patients were asked to rank all side-effects in this way whether they had experienced them or not.

Correspondence: EJ Maher, Mount Vernon Centre For Cancer Treatment, Rickmansworth Road, Northwood, Middlesex HA6 2RN, UK.
Received 6 December 1994; revised 14 August 1995; accepted 17 August 1995

However, some patients were not willing to rank side-effects they had not experienced. For consistency therefore, and because it seemed rational that information regarding acute side-effects actually experienced would be most meaningful, in analysing the acute morbidity data, only the symptoms or problems each patient had experienced were included in the importance ranking. For late morbidity, because numbers of patients actually suffering the complications were small, as long as patients had ranked every side-effect (whether they had experienced it or not) their ranking was included for analysis. A median importance rank across all patients was determined and the percentage of patients ranking each side-effect first, second or third in importance calculated for each symptom or complication.

For the purpose of part of this analysis, patients were divided into three groups on the basis of their overall treatment-response experiences, the hypothesis being that entirely positive, 'successful' treatment experiences, as opposed to those with a negative or 'unsuccessful' component, may influence future impressions of the 'preferred' treatment option. Thus, patients who had completely responded to initial treatment (either single modality or CMT) and who had maintained a complete response (CR) were defined as the 'successful' group and their answers were compared with those who had experienced 'unsuccessful' treatment scenarios. This second group included patients that had either, (a) gone into CR with first-line therapy but who had later relapsed requiring further treatment and, (b) those who had not achieved a CR with the first course of treatment and who went onto a second treatment regimen that had not been planned at the outset.

The whole of the first questionnaire was piloted on a group of ten patients to test for ambiguity and then re-piloted by circulation at a Hodgkin's Disease Association Annual General Meeting (Maher et al., 1990). Some of the questions were reformatted before sending further questionnaires.

The second questionnaire focused in more detail on both physical and psychosocial changes following treatment. Questions were based on those derived by Fobair et al., (1986) to evaluate potential disruptions in family and intimate relationships, sense of well-being and employment. Most of these data will not be presented here. Of direct relevance to this study were data concerning a number of early and late morbidities. In particular, patients' responses to questions regarding energy levels and fertility have been examined more closely to highlight issues relevant to data collection in clinical trials.

Simple counts, proportions, chi-squared and analysis of variance (ANOVA) were carried out on an Apple Macintosh using Statview (Statview, 1992).

### Results

### Information and involvement

In response to the question regarding the adequacy of information given, 80 patients (48%) felt they had not had enough information, 85 (51%) thought they had as much as they needed and no patients felt they had been given too much information. Fifty-one patients received written information about Hodgkin's disease and its treatment to take away with them and of the 113 that did not, 102 patients (90%) said they would have appreciated this.

The perceived level of patient involvement in the decision-making process for all patients is shown in Table I. A total of 102 patients (62%) indicated that they took no part in decision-making and that the doctor had been responsible for making all decisions on their behalf. Table II indicates that 95 patients (58%) were involved as much as they had wanted to be and the remainder were either dissatisfied with their level of involvement, could not remember or did not answer the question. Overall, patients having 'successful' and 'unsuccessful' treatment experiences (as defined previously) were equally likely to feel involved and satisfied with the decision-making process. Despite reporting that they had been adequately involved in the decision-making process, 25% of patients felt that there had been no real treatment choices available to them.

Table III shows the relationship between patients' perceptions of the adequacy of information given at or around the time of diagnosis and satisfaction with the level of involvement in the decision-making process. Patients who felt that they had been given sufficient information were statistically significantly more likely to have felt satisfied with their particular level of involvement, irrespective of their perceived degree of participation (or lack thereof). Sixty-seven of 77 patients (87%) happy with the information given were satisfied with their level of involvement compared with 10 of 77 (13%) who were not ($P<0.001$; $\chi^2$ test). Twenty-eight of 71 patients (39%) dissatisfied with the information given

**Table I** Perceptions of involvement in initial decision-making process in relation to experience of treatment response ($n = 165$).

| | *Successful* | *(%)* | *Unsuccessful* | *(%)* | *Total* |
|---|---|---|---|---|---|
| No involvement-all decisions made by doctor | 66 | (63) | 36 | (60) | 102 |
| Participated but felt that no real choices available | 25 | (24) | 16 | (27) | 41 |
| Participated and choices available | 6 | (6) | 6 | (10) | 41 |
| Cannot remember/did not answer | 8 | | 2 | | 10 |
| | 105 | | 60 | | 165 |

**Table II** Satisfaction with level of involvement in decision-making according to treatment experience ($n = 165$).

| | *Successful* | *(%)* | *Unsuccessful* | *(%)* | *Total* |
|---|---|---|---|---|---|
| As much involvement as desired | 56 | (53) | 39 | (65) | 95 |
| Less involved than would have liked | 35 | (33) | 18 | (20) | 53 |
| Cannot remember/ did not answer | 14 | | 3 | | 17 |
| | 105 | | 60 | | 165 |

were happy with the degree to which they were involved and the remaining 43 (61%) were not.

The 'success' or otherwise of the treatment experience did not appear to have any significant influence on this information/involvement relationship (ANOVA).

*Perceptions of acute and long-term morbidity*

The median ranking for each early side-effect listed in the questionnaire, the percentage of patients ranking it first, second or third and the number of patients experiencing the side-effect in question are shown in Table IV. Nausea and vomiting (with a median rank of 1 out of 11 items) was the side-effect viewed by patients as being the most important cause of acute morbidity to be considered when choosing between treatments with equal chance of cure. Ninety-three per cent of 122 patients experiencing this side-effect ranked it first, second or third. Change in physical appearance caused by weight gain and/or hair loss, pain, fatigue during treatment and sore mouth or difficulty swallowing all received a median importance ranking of 3.5 or greater and were experienced by 115, 56, 120 and 82 patients respectively. Weight gain from steroids was a much more commonly reported cause for concern in changed physical appearance than alopecia. For each of these four symptoms, between 50% and 62% of patients experiencing them ranked them first, second or third in terms of their influence on choice of further treatment. Septic episodes requiring admission, though only suffered by 19 patients, were felt by these patients to be very significant with a median ranking of 2; 68% of these patients ranked serious infection in the top three positions.

Of possible late complications, development of a second cancer, relapse of Hodgkin's disease and cardiovascular complications were thought to be the most important potential problems to be considered when choosing between different therapies, although numbers of patients experiencing many of these problems were relatively small (Table V). The percentage of patients ranking disease relapse requiring further treatment as first, second or third in importance in the late side-effect ranking was similar for the whole group of patients (80%) as compared with the group of patients actually experiencing a relapse of their disease (81%).

Data collected in the second questionnaire showed that 53 (32%) patients reported infertility following treatment. However, in the first questionnaire, only 21 (13%) patients ranked this potential complication as first, second or third in importance relative to other late effects. Of 48 (out of 53) patients reporting and ranking infertility, 15 of these ranked it first, second or third compared with only 6 of 44 patients who reported no fertility problems but who still ranked this late side-effect. This difference was statistically significant ($P = 0.05$). Thirty-five patients had had children (25 for the first time) after treatment and in only nine cases did patients feel 'it may be difficult/impossible for me to have children because of my Hodgkin's disease or its treatment'.

In the ranking section, 127 (73%) reported suffering from fatigue as an acute side-effect of treatment. When asked in a slightly different manner in the second questionnaire if their disease or its treatment had caused any alteration in energy

**Table III** Relationship between satisfaction with information, involvement in decision-making and success (S) or failure (F) of initial treatment

| | *Satisfaction with involvement* | | | | | |
|---|---|---|---|---|---|---|
| | *Yes* | | | *No* | | |
| Satisfaction with information | S | F | Total | S | F | Total |
| Yes | 41 | 26 | 67 | 6 | 4 | 10 |
| No | 15 | 13 | 28 | 29 | 14 | 43 |
| Total | 56 | 39 | 95 | 35 | 18 | 53 |

**Table IV** Importance ranking of acute side-effects

| *Potential side-effect* | *No. experiencing side-effect* | *Median rank* | *Percentage ranking 1, 2 or 3* |
|---|---|---|---|
| Nausea/vomiting | 122 | 1 | 93 |
| Serious infection | 19 | 2 | 68 |
| Appearance change | 115 | 3 | 62 |
| Pain | 56 | 3 | 59 |
| Fatigue | 120 | 3.5 | 50 |
| Sore mouth/throat | 82 | 3.5 | 50 |
| Cough/breathlessness | 103 | 5 | 5 |
| Numb hands/feet | 57 | 6 | 12 |
| Change in bowels | 56 | 6 | 16 |
| Loss of sex drive | 62 | 6.5 | 18 |
| Taste alteration | 90 | 7 | 8 |

**Table V** Late morbidity ranking by all patients (whether or not complication experienced) and number actually experiencing complication

| *Potential late complication* | *No. experiencing* | *No. ranking* | *Median rank* | *Percentage ranking 1, 2, 3* |
|---|---|---|---|---|
| Development of second cancer | 0 | 97 | 1 | 88 |
| Relapse of HD | 38 | 104 | 2 | 80 |
| Cardiovascular disease | 8 | 93 | 2.5 | 71 |
| Chronic energy loss | 48 | 104 | 5 | 18 |
| Infertility | 53 | 106 | 5 | 25 |
| Divorce/relationship problem | 21 | 92 | 5 | 21 |
| Anxiety/depression | 41 | 102 | 6 | 28 |
| Impaired sex life | 22 | 96 | 6 | 11 |
| Occupation/life insurance problem | 30 | 95 | 6 | 16 |

level, 156 patients (95%) said that energy levels had been reduced and in 104 (67%) of these, energy levels had remained decreased for several months after the treatment period; longer than 12 months in one-third of cases. Forty-six patients felt that their energy loss had never recovered up to the time of completing the questionnaire. In relation to influence on choice of therapy, patients gave long-term energy loss a median importance ranking of 5 (on a scale of 1–9). There was no significant difference between the incidences of energy reduction attached to differing treatment modalities, however post treatment energy reduction was slightly less common in patients receiving chemotherapy alone (56%) than those having radiotherapy alone (62%) or both treatment modalities (68%). The nature of the treatment experience, i.e. successful or unsuccessful, did not appear to impact on energy levels.

Of 76 patients receiving both chemotherapy and radiotherapy, 18 (24%) thought that the overall experiences of the two modalities were equally difficult, six that radiotherapy was more difficult and 52 (68%) that chemotherapy was worse than radiotherapy. Ninety-two of 152 patients answering the question (61%) thought that in general, when choosing between treatments with the same chance of cure, short-term, temporary side-effects in most people were most important while 23 (15%) thought permanent late problems occurring in a few people years after treatment were more important. Thirty-seven patients (24%) thought that acute and late side-effects were equally important in deciding choice of therapy.

### Discussion

There is increasing emphasis on patient autonomy and involvement in decision-making. This issue is particularly relevant in the management of early stage Hodgkin's disease in which there are often significant choices to be made between different treatment options. Many investigators have examined the concept of shared responsibility of patient care and of active patient participation in decision-making (Brody, 1980; Schain, 1980; Degner and Aquino Russell, 1988). This approach assumes that patients want to be actively involved in decision-making, an assumption refuted by some authors (Sutherland *et al.*, 1989; Fallowfield *et al.*, 1994).

Our data indicate that there are many patients who are satisfied with less than complete involvement, 26% of patients feeling satisfied with the doctor taking full responsibility for treatment decisions. Fifty-three patients (32%) had been involved at least partially in the decision-making process and the majority of these had been satisfied with this. It is not possible to be certain if satisfaction was influenced by the outcome of a therapy option chosen with or without the patient's input; that is whether a more favourable outcome (CR to initial treatment with no relapse) made the decision-making process seem more satisfying in retrospect than it actually was at the time, or would have seemed if the patient had relapsed and required further treatment. Our data imply, however, that treatment outcome has less influence on satisfaction with involvement and its relationship to information than might intuitively have been expected.

Doctors are demonstrably poor judges of patient preferences for involvement in their health care (Degner and Aquino Russell, 1988). Desire for involvement in management discussions and choices is affected by factors including patients' age (Cassileth *et al.*, 1980), sex (Blanchard *et al.*, 1988), cultural differences (Sensky, 1992) and time from cancer diagnosis. This last variable may be related to the stress around diagnosis inhibiting the ability to 'take on' information given or to seek further information for meaningful participation in management decisions, a problem reported by several authors. (Ley and Spelman, 1967; Degner and Aquino Russell, 1988). 'Prompt sheets' may facilitate patient participation in cancer consultations (Butow *et al.*, 1994). Furthermore, the severity of illness may influence desire for involvement. Ende *et al.* (1989) found that the more serious the disease, the less inclined patients are to participate in decisions. Strull *et al.* (1984) on the other hand reported that asymptomatic outpatients with essential hypertension similarly did not want an active role in management decisions.

Desire for active involvement in making choices regarding therapy is not well represented by a 'yes or no' model (Cassileth *et al.*, 1980). Nor does desire for participation necessarily mean that patients perceive real choices; 25% of our patients felt adequately involved without real choices being available. Several authors have dealt with the importance of how choices are presented (Strull *et al.*, 1984; Richards *et al.*, 1995). The reported benefits of providing choice (patient satisfaction, reduced stress, increased compliance, improved recovery) (Krantz *et al.*, 1980; Tuckett and Williams, 1984; Morris and Royle, 1988) must be balanced against the potentially increased anxiety associated with responsibility for decisions and regret for perceived 'wrong' decisions.

Another component of decision making is provision of information (Degner and Beaton, 1987). Patient satisfaction with health care has been directly linked to whether expectations of information from doctors are fulfilled (Degner and Aquino Russell, 1988). Others (Ley and Spelman, 1967; Ende *et al.*, 1989; Fallowfield *et al.*, 1994; Richards *et al.*, 1995) have stressed that desire for information is not necessarily an expression of a desire to be involved in primary decision-making. Ende *et al.* (1989) showed no correlation between patients' information seeking and decision-making preferences. Sutherland *et al.* (1989) found that many patients actively sought information, but the majority preferred the doctor to make treatment decisions. Like others (Cassileth *et al.*, 1980), Sutherland *et al.* demonstrate a positive correlation between degree of information seeking and level of preference for participation in decision-making. Our results suggest that the importance of adequate information may be in favourably influencing the overall experience of the decision-making process rather than necessarily encouraging participation in choosing treatments.

The amount and content of information given are only one aspect. How information is given may sometimes be more important than what is actually said (Tuckett and Williams, 1984). Some authors report that most patients asked directly express a desire for maximal information (Cassileth *et al.*, 1980), but suggest that too much information may increase stress, particularly at a time, e.g. soon after diagnosis, when recall and assimilation of information are especially impaired (Ley and Spelman, 1967; Blanchard *et al.*, 1988). The present study found that 90% of patients not receiving written information (62% of all patients) would have liked to have received written material. Although we did not specifically examine recall, these data would support the findings of others (Ley and Spelman, 1967), that in most groups of patients that less than half the information given was later recalled. Penman *et al.* (1984) suggested that, although written material was not as important as verbal information as a remembered source of information, it may have other values such as assisting communication between patients, oncologists, patients' relatives and other doctors.

It is increasingly recognised that overall or even disease-free survival are insufficient end points by which to compare treatment strategies in Hodgkin's disease. Several investigators (Fobair *et al.*, 1986; Newall *et al.*, 1987; Bloom *et al.*, 1993; Olweny *et al.*, 1993; Zeltzer, 1993) have examined the adaption of survivors of Hodgkin's disease and stress the importance of quality survival from both psychological and physical points of view. The emphasis in this study was to investigate the relative importance of different morbidities to give guidance to the content of initial information 'packages'.

Nausea and vomiting were perceived as the most problematic acute side-effects, as reported by others (Coates *et al.*, 1983)). The introduction of 5HT antagonists may have some bearing on this finding, although recent studies suggest that this remains a problem. The significance of septic episodes to those who had actually suffered them highlight the difficulty in conveying potential morbidities to people without experience of them. Symptoms assumed to be relatively minor, such as sore mouth and change in weight, loomed large in the patients' view. Similarly, energy loss was viewed as a considerable problem. Energy loss as a cause of late morbidity is rarely formally evaluated although the persistence of fatigue into the longer term has been repeatedly reported (Fobair *et al.* 1986; Bloom *et al.*, 1990, 1993) and was noted by 67% of patients in the current series. It was ranked at least equal (if not greater) in importance to other late psychosocial and physical complications with the exception of relapse of Hodgkin's disease or development of a second cancer or heart disease.

Infertility usually receives much attention and is often cited as a reason for recommending one treatment regimen over another. Clearly, this issue is crucial to many young patients. However results of the current study do suggest that this may be overemphasised when viewed from the perspective of the whole patient group. In this series, in only nine cases (5%) was Hodgkin's disease or its treatment felt to have rendered them infertile or potentially infertile when they might otherwise have had children. It should be noted that the British National Lymphoma Investigation philosophy at that time emphasised local therapy, at the cost of a slightly higher risk of relapse, more than some other strategies. This may have assisted in minimising the fertility issue, although infertility was still reported by a relatively large proportion of patients (32%). Zeltzer (1993) found that in young adults, concern regarding fertility following treatment was restricted to women. Among Hodgkin's disease patients treated at Stanford University, 78 of the 165 patients wanting to conceive following treatment (19% of all cases interviewed) were unable to do so (Fobair *et al.*, 1986). Kornblith *et al.* (1992) examined fertility in advanced Hodgkin's disease patients following treatment using an infertility index based on whether the patients believed they were or had been proven to be infertile or not. They found that although 53% were or believed they were infertile, this parameter was actually not a good predictor of survivors' overall psychological distress and concluded that it is in fact not a critical issue affecting long-term adjustment in Hodgkin's disease patients.

Our original hypothesis was that patients would rank risks of long-term side-effects of more importance than short-term, self-limiting and acute effects, but interestingly, patients perceived early acute side-effects as being at least as influential in determining choice of therapy as late, permanent morbidity despite the fact that almost exactly equal numbers of patients had some experience of early and late morbidity: 78% *vs* 75% for early and late side-effects respectively. It is a good example, however, of how difficult it can be to guess what patients' priorities are likely to be; a fact reported formally elsewhere. Zeltzer (1993) found similarly in a study of adolescent and young adult cancer survivors that acute side-effects of treatment were often the most vividly remembered and were reported as the worst aspect of having cancer. This must be acknowledged when offering increasingly intense initial therapies.

The issues raised by this study do not necessarily assist doctors in approaching the decision-making process for an individual patient. It must be acknowledged that cognitive dissonance affects patients' perception of the past; however, experienced surrogates have nonetheless given us counter-intuitive insights into the experience, preferences and priorities of patients. One solution to the problems in describing priorities might be to involve survivors in the design of clinical trials, or in the initial information-giving process. This approach has proved promising in other areas (Bradburn *et al.*, 1995). These data suggest that although many patients do wish for maximal participation in decision-making, many patients would prefer the doctor to take full responsibility for choice of therapy. Desire for information was more universal and related to satisfaction with the entire decision-making process. Desire for information and desire for full participation in decision-making can not be equated.

Further, these data support the notion that doctors treating Hodgkin's disease patients and designing trials in which to enter them may not necessarily have identified the outcomes that are of significance to patients themselves. It appears that acute side-effects may be of as least as much importance to patients as risk of late morbidity, and that early and late effects not routinely examined such as energy loss or change in appearance, may be more significant to patients than others that are regularly examined. If we are going to address the issues most pertinent to patients in this and other areas of oncology then the relevant questions must be incorporated into the design of future clinical trials. The results of this study further emphasise the need for routine use of validated self-report quality of life instruments.

**Acknowledgements**
Our sincere thanks to the Hodgkin's Disease Association, Cancer Research and the Cancer Research Campaign.

### References

BLANCHARD CG, LABRECQUE MS, RUCKDESCHEL JC AND BLANCHARD EB. (1988). Information and decision-making preferences of hospitalized adult cancer patients. *Soc. Sci. Med.*, **27,** 1139–1145.

BLOOM J, GORSKY R, FOBAIR P, HOPPE R, COX RS, VARGHESE A AND SPIEGAL D. (1990). Physical performance at work and at leisure: validation of a measure of biological energy in a sample of cancer survivors. *J. Psychosoc. Oncol.*, **6,** 49–63.

BLOOM JR, FOBAIR GE, WELLISCH D, SPIEGEL D, VARGHESE A AND HOPPE R. (1993). Psychosocial outcomes of cancer: a comparative analysis of Hodgkin's disease and testicular cancer. *J. Clin. Oncol.*, **11,** 979–988.

BRADBURN J, MAHER EJ, ADEWUYI-DALTON R, GRUNFELD E, LANCASTER T AND MANT D. (1995). Developing clinical trial protocols: the use of patient focus groups. Psycho-Oncology. *Psychol Oncol.*, **4:** 107–112.

BRODY DS. (1980). The patient's role in clinical decision-making. *Ann. Int. Med.*, **93,** 718–722.

BUTOW PN, DUNN SM, TATERSALL MHN AND JONES QJ. (1994). Patients participation in the cancer consultation: evaluation of a question prompt sheet; *Ann. Oncol.*, **5,** 199–204.

CASSILETH BR, ZUPKIS RV, SUTTON-SMITH K AND MARCH V. (1980). Information and participation preferences among cancer patients. *Ann. Int. Med.*, **92,** 832–836.

CLIMINO G, BITI GP, CARTONI C AND MAGRINI SM. (1992). Chemotherapy and radiotherapy in early stage Hogkins' disease : evidence of a more difficult rescue for patients relapsed after chemotherapy. *Eur. J. Cancer*, **11,** 1853–1859.

COATES A, ABRAHAMS S, KAYE SB, SOWERBUTTS T, FREWIN C, FOX RM AND TATTERSALL MH. (1983). On the receiving end–patient perception of the side-effects of cancer chemotherapy. *Eur. J. Cancer Clin. Oncol.*, **19,** 203–208.

CRNKOVICH MJ, LEOPOLD K, HOPPE RT AND MAUCH PM. (1987). Stage I to IIB Hodgkin's disease. The combined experience at Stanford University and the Joint Centre for Radiation Therapy. *J. Clin. Oncol.*, **5,** 1041–1049.

DEGNER LF AND AQUINO RUSSELL C. (1988). Preferences for treatment control among adults with cancer. *Res. Nursing Health*, **11,** 367–374.

DEGNER LF AND BEATON JI. (1987). *Life–death Decisions in Health Care*. Hemisphere: Washington.

ENDE J, KAZIS L, ASH A AND MOSKOWITZ MA. (1989). Measuring patients' desire for autonomy: decision making and information-seeking preferences among medical patients. *J. Gen. Int. Med.*, **4,** 23–30.

FARAH R, ULTMANN J, GRIEM M, GOLOMB H, KALOKHE V, DESSER R, BLOUGH R AND WEICHSELBAUM R. (1988). Extended mantle radiation therapy for pathologic stage I and II Hodgkin's disease. *J. Clin. Oncol.*, **6**, 1047-1058.

FALLOWFIELD LJ, HALL A, MAGUIRE P, BAUM M AND A'HERN RP. (1994). Psychological effects of being offered choice of surgery for breast cancer. *Br. Med. J.*, **309**. 448.

FOBAIR P, HOPPE RT, BLOOM JR, COX R, VARGHESE A AND SPIEGEL D. (1986). Psychosocial problems among survivors of Hodgkin's disease. *J. Clin. Oncol.*, **4**, 805-814.

KORNBLITH AB, ANDERSON J, CELLA DF, TROSS S, ZUCKERMAN E, CHERIN E, HENDERSON E, WEISS RB, COOPER R, SILVER RT, LEONE L, CANELLOS GP, GOTTLIEB A AND HOLLAND JC. (1992). Hodgkin Disease survivors at increased risk for problems in psychosocial adaption. *Cancer*, **70**, 2214-2223.

KRANTZ DS, BAUM A AND WIDEMAN MV. Assessment of preferences for self-treatment and information in health care. (1980). *J. Personal Soc. Psychol.*, **39**, 977-990.

LEY P AND SPELMAN MS. (1967). *Communicating with the Patient*. Staples Press: London.

MORRIS J AND ROYLE GT. (1988). Offering patients a choice of surgery for early breast cancer: a reduction in anxiety and depression in patients and their husbands. *Soc. Sci. Med.*, **26**, 583-585.

NEWALL DJ, GADD EM AND PRIESTMAN TJ. (1987). Presentation of information to cancer patients: a comparison of two centres in the UK and USA. *Br. J. Med. Psychol.*, **60**, 127-131.

OLWENY CLM, JUTTNER CA, ROFE P, BARROW G, ESTERMAN A, WALTHAM R, EHTESHAM A, CHESTERMAN H, SESHADRI R, SAGE E, ANDARY C, KATSIKITIS M, ROBERTS M AND SELVANAYAGAM S. (1993). Long-term effects of cancer treatment and consequences of cure: cancer survivors enjoy quality of life similar to their neighbours. *Eur. J. Cancer*, **29A**, 826-830.

PENMAN DT, HOLLAND JC, BAHRA GF, MORROW G, SCHMALE AH, DEROGATIS LR, CARNIKE CL AND CHERRY R. (1984). Informed consent for investigational chemotherapy: patients' and physicians' perceptions. *J. Clin. Oncol.*, **2**, 849-855.

RICHARDS MA, RAMIREZ AJ, DEGNER LF, FALLOWFIELD LJ, MAHLER EJ AND NEUBERGER RJ. (1995). Offering choice of treatment to patients with cancers. A review based on a Symposium held at the 10th Annual Conference of the British Psychosocial Oncology Group, December 1993. *Eur. J. Cancer*, **31A**, 112-116.

SENSKY T. (1992). Asking patients about their treatment. Why their answers should not always be taken at face value. *Br. Med. J.*, **305**, 1109-1110.

SCHAIN WS. (1980). Patients' rights in decision-making: the case for personalism versus paternalism in health care. *Cancer*, **46**, 1035-1041.

SLEVIN ML, STUBBS L, PLANT HJ, WILSON P, GREGORY WM, ARMES PJ AND DOWNER SM. (1990). Attitudes to chemotherapy comparing views of patients with cancer with those of doctors, nurses, and general public. *Br. Med. J.*, **300**, 1458-1460.

STATVIEW. (1992). Abacus Concepts, Berkeley, USA.

STRULL WM, BERNARD L AND CHARLES G. (1984). Do patients want to participate in medical decision making? *JAMA*, **252**, 2990-2994.

SUTHERLAND HJ, LLEWELLYN-THOMAS HA, LOCKWOOD GA, TRITCHLER DL AND TILL JE. (1989). Cancer patients: their desire for information and participation in treatment decisions. *J. R. Soc. Med.*, **82**, 260-263.

TUCKETT D AND WILLIAMS A. (1984). Approaches to the measurement of explanation and information-giving in medical consultations: a review of empirical studies. *Soc. Sci. Med.*, **18**, 571-580.

ZELTZER LK. (1993). Cancer in adolescents and young adults. Psychosocial aspects. *Cancer*, **71**, (suppl.). 3463-3468.